# Supplementary material for: Delftia acidovorans secretes substances that inhibit the growth of Staphylococcus epidermidis through TCA cycle-triggered ROS production
Source: PLoS One. 2021 Jul 2;16(7):e0253618. doi: 10.1371/journal.pone.0253618 (PMC8253425; doi:10.1371/journal.pone.0253618)
Supplement: S1 File — (DOCX) [file pone.0253618.s001.docx]

***Delftia acidovorans* secretes substances that inhibit growth of *Staphylococcus epidermidis* through TCA cycle triggered-ROS production.**

Tomotaka Ohkubo^1, 2^, Yasuhiko Matsumoto^1^, Otomi Cho^1^, Yuki Ogasawara^2^, and, Takashi Sugita^1^*

^1^ Department of Microbiology, Meiji Pharmaceutical University, Kiyose, Tokyo 204-8588, Japan.

^2^ Department of Analytical Biochemistry, Meiji Pharmaceutical University, Kiyose, Tokyo 204-8588, Japan.

* Corresponding author

E-mail: sugita@my-pharm.ac.jp (TS)

**Supplementary Table 1 Up-regulation of *S. epidermidis* genes by culture supernatant of *D. acidovorans***

| Locus name | Gene name | Protein function (Genbank) | Fold Change |
| --- | --- | --- | --- |
| SE1134 | *gpsB* | conserved hypothetical protein | 774.2 |
| SE1805 | *rpmD* | 50S ribosomal protein L30 | 120.0 |
| SE2159 |  | conserved hypothetical protein | 24.0 |
| SE0479 | *saeR* | response regulator | 13.7 |
| SE0756 | *qoxD* | putative quinol oxidase polypeptide IV QoxD | 12.7 |
| SE1425 |  | conserved hypothetical protein | 9.1 |
| SE2366 |  | hypothetical protein | 8.5 |
| SE0062 |  | copper-transporting ATPase copA | 7.6 |
| SE0157 | *mqo3* | malate:quinone oxidoreductase | 7.0 |
| SE2234 |  | hypothetical protein | 6.8 |
| SE2160 |  | phosphoenolpyruvate-protein phosphatase | 5.6 |
| SE1684 |  | conserved hypothetical protein | 5.5 |
| SE1259 |  | conserved hypothetical protein | 5.0 |
| SE1773 |  | alkaline shock protein 23 | 4.9 |
| SE1119 | *folA, dfrC, folA1* | dihydrofolate reductase | 4.9 |
| SE1361 | *gapA2 gapB* | glyceraldehyde 3-phosphate dehydrogenase 2 | 4.6 |
| SE1739 |  | conserved hypothetical protein | 4.6 |
| SE2069 |  | conserved hypothetical protein | 4.5 |
| SE1169 | *ebp* | elastin binding protein | 4.4 |

**Supplementary Table 1 (continued) Up-regulation of *S. epidermidis* genes by culture supernatant of *D. acidovorans***

| Locus name | Gene name | Protein function (Genbank) | Fold Change |
| --- | --- | --- | --- |
| SE1412 |  | general stress protein-like protein | 4.3 |
| SE1711 |  | conserved hypothetical protein | 4.3 |
| SE2072 |  | hypothetical protein | 4.2 |
| SE0389 |  | putative esterase/lipase | 4.2 |
| SE2286 |  | translation initiation inhibitor-like protein | 4.1 |
| SE0889 |  | conserved hypothetical protein | 4.0 |
| SE0729 |  | conserved hypothetical protein | 3.9 |
| SE0560 | *gpmI* | phosphoglycerate mutase | 3.7 |
| SE1763 |  | conserved hypothetical protein | 3.7 |
| SE1774 | *asp23* | alkaline shock protein 23 | 3.6 |
| SE1775 |  | conserved hypothetical protein | 3.6 |
| SE0553 |  | cell-division inhibitor | 3.6 |
| SE0074 |  | putative divalent heavy-metal cations transporter | 3.5 |
| SE0110 |  | conserved hypothetical protein | 3.5 |
| SE0554 |  | conserved hypothetical protein [ | 3.5 |
| SE1017 | *rpmG2* | 50S ribosomal protein L33 | 3.5 |
| SE0341 |  | putative hexulose-6-phosphate synthase | 3.5 |
| SE2173 |  | hypothetical protein | 3.4 |
| SE1174 |  | conserved hypothetical protein | 3.4 |

**Supplementary Table 1 (continued) Up-regulation of *S. epidermidis* genes by culture supernatant of *D. acidovorans***

| Locus name | Gene name | Protein function (Genbank) | Fold Change |
| --- | --- | --- | --- |
| SE0367 |  | oxidoreductase ion channel | 3.3 |
| SE0080 |  | regulatory protein | 3.3 |
| SE0558 | *pgk* | phosphoglycerate kinase | 3.3 |
| SE1962 |  | general stress protein 26 | 3.2 |
| SE1785 | *lacC* | tagatose-6-phosphate kinase | 3.2 |
| SE0585 |  | conserved hypothetical protein | 3.1 |
| SE0855 | *ftsL* | cell division protein | 3.1 |
| SE1560 |  | conserved hypothetical protein | 3.1 |
| SE1777 |  | alginate lyase | 3.1 |
| SE0557 | *gapA1, gap, gapA* | glyceraldehyde-3-phosphate dehydrogenase | 3.1 |
| SE2351 |  | conserved hypothetical protein | 3.1 |
| SE2287 |  | PurR | 3.0 |
| SE2191 |  | conserved hypothetical protein | 2.9 |
| SE1708 |  | UDP-GlcNAc 2-epimerase | 2.9 |
| SE0861 | *ftsZ* | cell division protein | 2.9 |
| SE1382 |  | conserved hypothetical protein | 2.9 |
| SE0559 | *tpiA* | triosephosphate isomerase | 2.9 |
| SE2103 |  | pyruvate oxidase | 2.9 |
| SE1240 | *sodA* | superoxide dismutase SodA | 2.8 |

**Supplementary Table 1 (continued) Up-regulation of *S. epidermidis* genes by culture supernatant of *D. acidovorans***

| Locus name | Gene name | Protein function (Genbank) | Fold Change |
| --- | --- | --- | --- |
| SE1738 |  | general stress protein 20U | 2.8 |
| SE0591 |  | conserved hypothetical protein | 2.8 |
| SE1970 | *nreB* | two component sensor histidine kinase | 2.7 |
| SE0439 | *scdA* | cell division and morphogenesis-related protein | 2.7 |
| SE1409 | *ccpA* | catabolite control protein A | 2.7 |
| SE1309 |  | conserved hypothetical protein | 2.6 |
| SE0310 | *rpsG* | 30S ribosomal protein S7 | 2.6 |
| SE0472 |  | PTS system fructose-specific IIABC component | 2.6 |
| SE1519 |  | conserved hypothetical protein | 2.6 |
| SE1710 | *glyA* | serine hydroxymethyl transferase | 2.6 |
| SE0996 |  | conserved hypothetical protein | 2.5 |
| SE2306 |  | conserved hypothetical protein | 2.5 |
| SE0534 | *hpf* | conserved hypothetical protein | 2.5 |
| SE0805 |  | putative myo-inositol-1(or 4)-monophosphatase | 2.5 |
| SE1776 |  | conserved hypothetical protein | 2.5 |
| SE0197 | *butA* | acetoin(diacetyl)reductase | 2.5 |
| SE1016 | *katA* | Catalase | 2.5 |
| SE2285 | *spoVG* | spoVG protein | 2.5 |
| SE0586 |  | conserved hypothetical protein | 2.4 |
| SE0255 |  | branched-chain alpha-keto acid dehydrogenase E1 | 2.4 |

**Supplementary Table 1 (continued) Up-regulation of *S. epidermidis* genes by culture supernatant of *D. acidovorans***

| Locus name | Gene name | Protein function (Genbank) | Fold Change |
| --- | --- | --- | --- |
| SE1535 |  | conserved hypothetical protein | 2.4 |
| SE0258 |  | immunodominant antigen B | 2.4 |
| SE1384 | *ald* | alanine dehydrogenase | 2.4 |
| SE1790 | *rpsI* | 30S ribosomal protein S9 | 2.4 |
| SE2145 | *ldh* | L-lactate dehydrogenase | 2.4 |
| SE1206 |  | conserved hypothetical protein | 2.4 |
| SE0658 | *pgi* | glucose-6-phosphate isomerase A | 2.3 |
| SE0757 | *qoxC* | quinol oxidase polypeptide III QoxC | 2.3 |
| SE1116 | *msrB* | conserved hypothetical protein | 2.3 |
| SE1381 |  | conserved hypothetical protein | 2.3 |
| SE0448 |  | conserved hypothetical protein | 2.2 |
| SE1272 | *rpsT* | 30S ribosomal protein S20 | 2.2 |
| SE1971 |  | conserved hypothetical protein | 2.2 |
| SE0665 |  | 5-oxo-1,2,5-tricarboxilic-3-penten acid decarboxylase | 2.2 |
| SE1371 |  | citrate synthase II | 2.2 |
| SE2251 |  | hypothetical protein | 2.2 |
| SE2144 |  | acetolactate synthase large subunit | 2.1 |
| SE0688 | *mecA* | negative regulator of genetic competence MecA | 2.1 |
| SE1781 | *lacG* | 6-phospho-beta-galactosidase | 2.1 |
| SE1370 | *icd citC* | isocitrate dehydrogenase | 2.1 |

**Supplementary Table 1 (continued) Up-regulation of *S. epidermidis* genes by culture supernatant of *D. acidovorans***

| SE1268 | *grpE* | GrpE protein | 2.1 |
| --- | --- | --- | --- |
| SE2367 |  | conserved hypothetical protein | 2.1 |
| SE1329 |  | conserved hypothetical protein | 2.0 |

**Supplementary Table 2 Down-regulation of *S. epidermidis* genes by culture supernatant of *D. acidovorans***

| Locus name | Gene name | Protein function (Genbank) | Fold Change |
| --- | --- | --- | --- |
| SE1548 | *hemL2, gsaB* | glutamate-1-semialdehyde aminotransferase | 0.15 |
| SE2014 | *lrgB* | holin-like protein LrgB | 0.17 |
| SE1093 |  | conserved hypothetical protein | 0.26 |
| SE0238 |  | conserved hypothetical protein | 0.35 |
| SE0237 |  | conserved hypothetical protein | 0.36 |
| SE2082 |  | regulatory protein pfoR | 0.49 |
| SE2081 |  | putative beta-subunit of L-serine dehydratas | 0.50 |


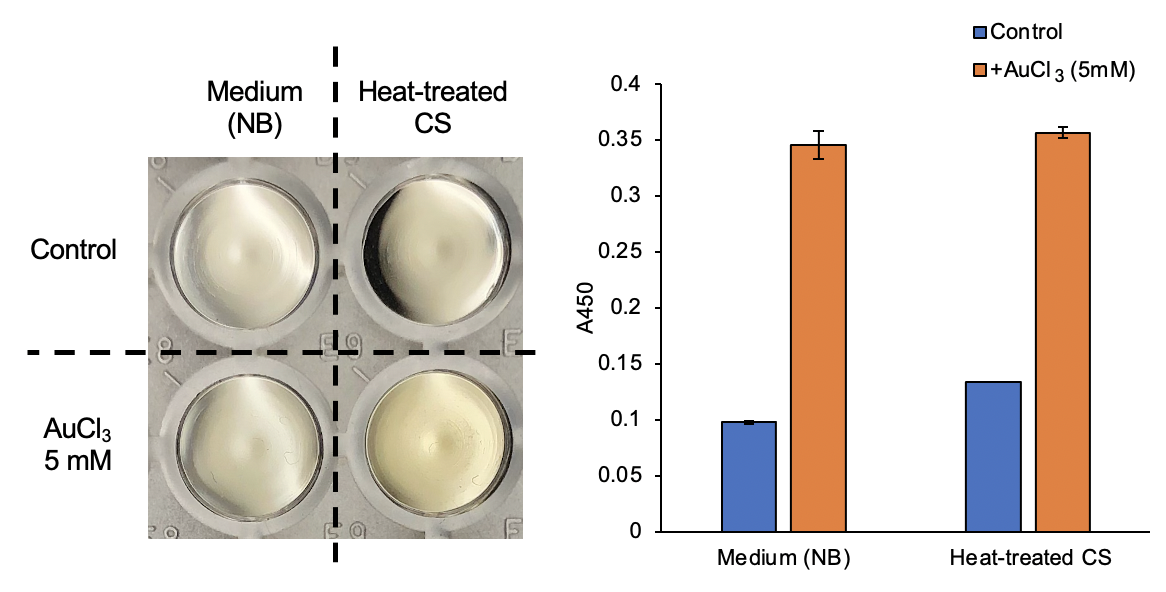


**Supplementary Figure 1 The heat-treated culture supernatant of *D. acidovorans* used in this study did not precipitate gold.**

Four microliters of 125 mM AuCl_3_ (Wako Pure Chemical Corporation, Osaka, Japan) was added to 96 μL of medium (NB) or *D. acidovorans* heat-treated CS (Heat-treated CS) placed in a 96-well plate (TPP, Schaffhausen, Switzerland). After incubating at 37℃ for 12 h, absorbance at 450 nm was measured using a microplate reader (iMark™ microplate reader; Bio-Rad Laboratories Inc., Hercules, CA, USA). Error bars indicate the SD of the means (n=3).


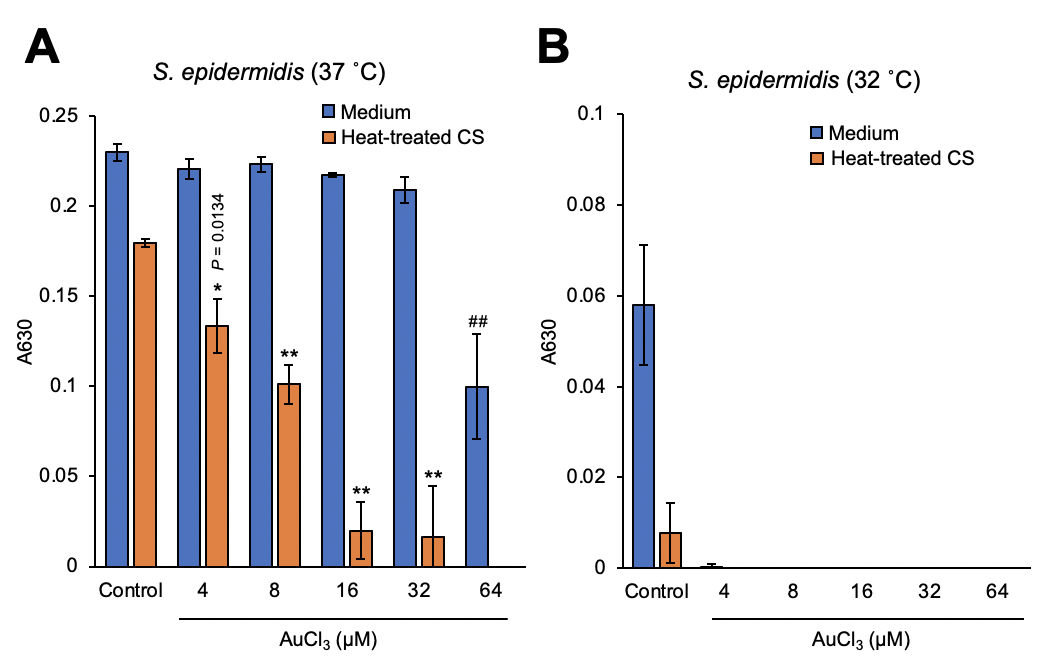


**Supplementary Figure 2 The heat-treated culture supernatant of *D. acidovorans* used in this study did not detoxify soluble gold.**

*D. acidovorans* heat-treated CS (Heat-treated CS) and AuCl_3_ (Wako Pure Chemical Corporation, Osaka, Japan) were diluted with NB to 30% and 8-128 μM, respectively, and dispensed in 50-µL aliquots into a 96-well plate (TPP). *S. epidermidis* (2 x 10^4^ cells/ml) was prepared with NB and 50 µL was added to each well. After incubating at 37℃ for 24 h (A) or 32℃ for 28 h (B), absorbance at 630 nm was measured using a microplate reader (iMark™ microplate reader; Bio-Rad Laboratories Inc.). Error bars indicate the SD of the means (n=3). Statistical differences between groups were analyzed by the Dunnett test. (^#^ : *P* < 0.05 (Medium), ^# #^：*P* < 0.001 (Medium), *： *P* < 0.05 (Heat-treated CS), **：*P* < 0.001(Heat-treated CS))
