# Supplementary material for: Delftia acidovorans secretes substances that inhibit the growth of Staphylococcus epidermidis through TCA cycle-triggered ROS production
Source: PLoS One. 2021 Jul 2;16(7):e0253618. doi: 10.1371/journal.pone.0253618 (PMC8253425; doi:10.1371/journal.pone.0253618)
Supplement: Attachment — Submitted filename: Response to Reviewers.doc [file pone.0253618.s003.doc]

Response to reviewer’s comments

1. Is the manuscript technically sound, and do the data support the conclusions?

The manuscript must describe a technically sound piece of scientific research with data that supports the conclusions. Experiments must have been conducted rigorously, with appropriate controls, replication, and sample sizes. The conclusions must be drawn appropriately based on the data presented.

Reviewer #1: Partly

Reviewer #2: Yes

We now state in the revised manuscript that all experiments were replicated at least twice, as follows: "All experiments were performed at least twice." (Materials & Methods section, page 10, line 148)

The sample sizes for each experiment are provided in the figure legends of the revised manuscript (pages 24-27, lines 406-472).

2. Has the statistical analysis been performed appropriately and rigorously?

Reviewer #1: Yes

Reviewer #2: Yes

Thank you very much for reviewing our manuscript.

3. Have the authors made all data underlying the findings in their manuscript fully available?

The [PLOS Data policy](http://www.plosone.org/static/policies.action#sharing) requires authors to make all data underlying the findings described in their manuscript fully available without restriction, with rare exception (please refer to the Data Availability Statement in the manuscript PDF file). The data should be provided as part of the manuscript or its supporting information, or deposited to a public repository. For example, in addition to summary statistics, the data points behind means, medians and variance measures should be available. If there are restrictions on publicly sharing data—e.g. participant privacy or use of data from a third party—those must be specified.

Reviewer #1: No

Reviewer #2: No

We added the S1 Dataset file, which includes raw data related to our results, to the supplementary information of the revised manuscript (page 17, lines 262-264). Moreover, our data related to the RNA sequence analysis have been deposited at DDBJ/ENA/GenBank: Submission ID = mpu_microbiology-0001, BioProject = PRJDB11464 BioSample =SAMD00294166, SAMD00294165, Accession number = DRA011812. A data availability statement including this information has been added to the revised manuscript (page 17, lines 262-267).

4. Is the manuscript presented in an intelligible fashion and written in standard English?

PLOS ONE does not copyedit accepted manuscripts, so the language in submitted articles must be clear, correct, and unambiguous. Any typographical or grammatical errors should be corrected at revision, so please note any specific errors here.

Reviewer #1: No

Reviewer #2: Yes

Following the referee’s comment, the revised manuscript was edited by professional native English-speaking science editors (SciTechEdit International, LLC, CO, USA).

5. Review Comments to the Author

Reviewer #1: Dear authors,

You mentioned atopic dermatitis in your manuscript. However, the relation between your findings and atopic dermatitis is unclear. For example, in other inflammatory skin disorders, such as psoriasis, the skin microbiome will be changed as well. in other words, even though your findings may also be applicable to other skin disorders, there is no such discussion in your manuscript and/or the discussion of the relevance and significance of your findings with regards to atopic dermatitis is lacking. So please clarify your hypothesis and provide a discussion of the results and importance of your findings in atopic dermatitis. English needs to be edited.

According to the reviewer’s comment, we now mention the relationship between the skin microbiome and skin disorders such as psoriasis and acne vulgaris in the Introduction and Discussion sections of the revised manuscript (pages 4-5, lines 53-56; pages 15-16, lines 227-233).

Pages 4-5, lines 53-56.

"The relative abundance of *S. aureus* is increased in the skin of patients with psoriasis (PS) as well as in those with AD. *S. epidermidis* also inhibits the growth of *Cutibacterium acnes,* which is associated with acne vulgaris (AV). Therefore, *S. epidermidis* might play a key role in various skin disorders."

Pages 15-16, lines 227-233.

"In the skin of patients with AD, the relative abundance of *S. aureus* is significantly increased, and enterotoxin or protease derived from *S. aureus* exacerbates inflammation of AD. The relative abundance of *S. aureus* is also increased in the skin of patients with PS and Th17 cytokines are induced by *S. aureus. S. epidermidis* inhibits the growth of *S. aureus,* which might be associated with AD and PS. Moreover, *S. epidermidis* inhibits the growth of *C. acnes,* which is associated with AV."

We also added our hypothesis and provide a discussion of the results and importance of our findings in relation to AD in the revised manuscript (page 16, lines 233-237).

Page 16, lines 233-237.

“We therefore hypothesized that the inhibitory activity of substances secreted by *D. acidovorans* against *S. epidermidis* is indirectly associated with exacerbation of AD, PS, and AV. *D. acidovorans* might be related to various skin disorders. Additional studies are needed to elucidate the relationship between *D. acidovorans* and AD, PS, and AV."

Following the referee’s comment, the revised manuscript was edited by professional native English-speaking science editors (SciTechEdit International, LLC, CO, USA).

Reviewer #2: Rather than mentioning your findings in the end of introduction, why don’t you add the work-hypothesis or objectives? It will thus help the student-readers to have an overview of a typical research article. Moreover, the introduction should briefly be extended to better comprehend the study topic.

According to the reviewer’s comment, we added the following objective at the end of the Introduction section in the revised manuscript (page 5, lines 63-64).

Page 5 lines 63-64.

"In the present study, we investigated the effect of *D. acidovorans* on the growth of *S. epidermidis* and elucidated its mechanisms."

Does 100 ˚C heat treatment (for 30 min) to bacterial culture supernatant is ok for metabolites integrity?

The substances secreted by *D. acidovorans* may be heat-sensitive. On the other hand, we confirmed that the antimicrobial activity of substances secreted by *D. acidovorans* was not decreased by heat treatment at 100˚C for 30 min (Fig. 1 and Fig. 2). Therefore, we assumed that substances secreted by *D. acidovorans* are stable to 100˚C heat treatment for at least 30 min.

What about the stability of the chemicals?

Because *D. acidovorans* CS exhibits antimicrobial activity after heat treatment at 100˚C, we assumed that substances secreted by *D. acidovorans* are heat-stable (Fig. 1 and Fig. 2).

In addition, we confirmed that the culture supernatant of *D. acidovorans* stored at 4˚C for 1 year exhibits the same amount of antimicrobial activity as a fresh sample. Therefore, we assumed that the substances were also stable in nutrient broth.

I would appreciate if the authors may provide the rationale and appropriate literature references for this temperature treatment. And, what were the heat stable substance in this treated culture?

According to the reviewer’s comment, we added a sentence to the Discussion section of the revised manuscript (page 14, lines 203-205).

Page 14, lines 203-205.

“A previous study demonstrated that *D. acidovorans* secreted heat-stable antimicrobial substances that inhibit the growth of *S. aureus* (Tejman-Yarden N., *et al*., Front Microbiol. 10:2377, 2019). Therefore, we assessed the inhibitory activity of *D. acidovorans* heat-treated CS against *S. epidermidis.*“

The active substance has not yet been identified. We added the following sentence to the Discussion section of the revised manuscript (page 17, lines 252-254).

Page 17, lines 252-254.

“Identifying the active substances in the *D. acidovorans* heat-treated CS is an important topic for future studies.”

Why D. acidovorans JCM6218 strain was grown at 27℃ and subsequently on 32℃, if this bacterium is usually present on skin surfaces?

A previous study reported that *D. acidovorans*, a common environmental bacteria, can be grown at 4–41˚C (Wen A, *et al*., Int J Syst Bacteriol. 2:567-76, 1999). In addition, we confirmed that *D. acidovorans* JCM6218 can also be grown at 27˚C without any problem. Therefore, we incubated the *D. acidovorans* JCM6218 strain at 27˚C from stock before using it to perform the assay at 32˚C (temperature of human skin).

Reference：A Wen et al. Int J Syst Bacteriol. 2:567-76, 1999. doi: 10.1099/00207713-49-2-567.

Line-153 says that D. acidovorans heat-treated CS did not decrease the viable number of S. epidermis (Fig 3); while Line 155-6 say that heat-stable substances secreted by D. acidovorans have bacteriostatic activity against S. epidermidis. Please clarify the potential contradiction.

According to the reviewer’s comment, we added sentences about the bacteriostatic activity of the *D. acidovorans* CS in the Results section of the revised manuscript (page 11, lines 158-164).

Chemicals such as antibiotics with bacteriostatic activity inhibit the growth of a bacterium, but do not kill cells. On the other hand, chemicals with bactericidal activity inhibit the growth of a bacterium and kill the cells.

We examined whether the *D. acidovorans* heat-treated CS had bactericidal activity against *S. epidermidis* in phosphate buffered saline (PBS). *S. epidermidis* cannot grow in PBS, but can survive at least 12 h. Gentamicin was used as a positive control because it exhibits bactericidal activity. The addition of gentamicin decreased the number of viable bacteria within 3 h, whereas the addition of *D. acidovorans* heat-treated CS had no effect (Fig 3). The *D. acidovorans* heat-treated CS, however, has the potential to inhibit the growth of *S. epidermidis* in a nutrient broth. Because the *D. acidovorans* heat-treated CS, which can inhibit the growth of *S. epidermidis* in a nutrient broth, did not kill the *S. epidermidis* cells, we assumed that the inhibitory activity of *D. acidovorans* heat-treated CS against *S. epidermidis* is bacteriostatic.

Page 11, lines 158-164.

“We next examined whether the inhibitory activity of *D. acidovorans* heat-treated CS against *S. epidermidis* is bactericidal or bacteriostatic. *D. acidovorans* heat-treated CS did not decrease the viable number of *S. epidermis* in PBS (Fig 3). On the other hand, gentamicin, which has bactericidal activity, decreased the number of viable bacteria within 3 h (Fig 3). These results suggest that heat-stable substances secreted by *D. acidovorans* exhibit bacteriostatic activity, but not bactericidal activity, against *S. epidermidis*.”

As per your data, 6-genes related to glycolysis and 3 genes related to the TCA cycle in S. epidermidis was increased more than 2-fold by the D. acidovorans CS ; and then malonic acid (4 mM) was used to verify the inhibitory effect via TCA cycle. I wonder why authors were convinced to verify their 3-genes TCA cycle and not 6-genes glycolysis? Please describe your rationale for this selection.

According to the reviewer’s comment, we added a sentence to the Results section of the revised manuscript (page 12, lines 176-179).

Because pyruvate produced by glycolysis triggers the TCA cycle, we speculated that induction of genes related to glycolysis leads to activation of the TCA cycle. In this study, genes of *S. epidermidis* related to both glycolysis and the TCA cycle were upregulated by *D. acidovorans* CS. Therefore, we focused on activation of the TCA cycle, which is downstream of glycolysis.

Page 12, lines 176-179.

"Because pyruvate produced by glycolysis triggers TCA cycle activation, we focused on the relationship between activation of TCA cycle, which is downstream of glycolysis, and the inhibitory activity of *D. acidovorans* heat-treated CS against *S. epidermidis*."

Why figure 7 data is presented in discussion and not in results section of the ms?

According to the reviewer’s comment, we moved Figure 7 to the Results section in the revised manuscript (Page 13, line 192).

Moreover, I suggest authors to further infer their results to furnish their discussion with more literature studies. For instance, authors could think of chemical substances other than delftibactin only which may inhibit the target bacterium in this case.

According to the reviewer’s suggestion, we added the following sentences to the Discussion section of the revised manuscript to describe that active substances besides delftibactin are possibly presented by *D. acidovorans* CS.

"Tejman-Yarden et al. indicated the existence of a fraction other than the delfibactin fraction that exerts antimicrobial activity in the culture supernatant of *D. acidovorans.* In addition, a substance characterized as C_39_H_68_N_14_O_17_, which is similar to the composition of delftibactin, is present in the delfibactin fraction. It is possible that the active substances focused on in this study are in these fractions. Identifying the active substances in the *D. acidovorans* heat-treated CS is an important topic for future studies." (pages 16-17, lines 248-254)

At present, I have the feeling that authors have just briefly reported their work; they could rather better describe their findings with sufficient discussion points and literature review in the introduction.

According to the reviewer’s suggestion, we added sentences to the Introduction and Discussion sections of the revised manuscript (pages 4-5, lines 53-56; page 5, lines 63-64; page 5, lines 67-70; pages 15-16, lines 227-237).

Page 4-5, lines 53-56.

" The relative abundance of *S. aureus* is increased in the skin of patients with psoriasis (PS) as well as in those with AD (Totté JEE, *et al*., Eur J Clin Microbiol Infect Dis, 35:1069–1077, 2016, Chang HW, *et al*., Microbiome, 6:154-27, 2018). *S. epidermidis* also inhibits the growth of *Cutibacterium acnes,* which is associated with acne vulgaris (AV) (Wang Y., *et al*., Appl Microbiol Biotechnol, 98:411-424, 2014, Christensen GJM, *et al*., BMC Genomics, 17:152-14, 2016, Nakamura K., *et al*., Sci Rep, 10:21237-12, 2020). Therefore, *S. epidermidis* might play a key role in various skin disorders."

Page 5, lines 63-64.

“In this present study, we investigated the effect of *D. acidovorans* on the growth of *S. epidermidis* and elucidated its mechanisms.”

Page 5, lines 67-70.

“Our findings provide important insight into how *D. acidovorans* affects the skin microbiome by inhibiting the growth of *S. epidermidis*, resulting in a skin microbiome imbalance related to various skin disorders.”

Page 15-16, lines 227-237.

" In the skin of patients with AD, the relative abundance of *S. aureus* is significantly increased, and enterotoxin or protease derived from *S. aureus* exacerbates inflammation of AD. The relative abundance of *S. aureus* is also increased in the skin of patients with PS and Th17 cytokines are induced by *S. aureus*. *S. epidermidis* inhibits the growth of *S. aureus,* which might be associated with AD and PS. Moreover, *S. epidermidis* inhibits the growth of *C. acnes,* which is associated with AV. We therefore hypothesized that the inhibitory activity of substances secreted by *D. acidovorans* against *S. epidermidis* is indirectly associated with exacerbation of AD, PS, and AV. *D. acidovorans* might be related to various skin disorders. Additional studies are needed to elucidate relationship between *D. acidovorans* and AD, PS, and AV. "

Figure legends are within the ms-text (and sometimes in interrogative case. I would suggest to keep the legends narrative, and all legends together after references.

According to the reviewer’s suggestion, we changed the location of the figure legends in the revised manuscript.

RNA seq data should be available on some public database and be cited in ms accordingly.

According to the reviewer’s suggestion, we deposited our raw data of the RNA seq analysis with the DDBJ database (Submission ID = mpu_microbiology-0001, BioProject = PRJDB11464, BioSample = SAMD00294166, SAMD00294165, Accession number = DRA011812). Moreover, we added a data availability statement section describing this in the revised manuscript (page 17, lines 262-267).

6. PLOS authors have the option to publish the peer review history of their article ([what does this mean?](https://journals.plos.org/plosone/s/editorial-and-peer-review-process#loc-peer-review-history)). If published, this will include your full peer review and any attached files.

We would like to publish the peer review history of this article.
